# Book Reviews

**Published:** 1905-04

**Authors:** 


A Textbook of Histology. By Frederick R. Bailey, A.M., M.D., Adjunct Professor of Normal Histology, College of Physicians and Surgeons, New York. Octavo, 481 pages. Illustrated. New York: William Wood & Company. 1904. (Price, $3.00.)

The fascinating study of histology is delineated in this book with great clearness. The chapters on technic are the most practical we have seen. The study of the cell, that simplest form of animal life, is made so attractive that one can scarcely resist the temptation to reënter the laboratory and begin over again, under the guidance of this book. After the cell the tissues are dealt with in six chapters, including the epithelium, the connective tissues, the blood, lymphatic, muscle, and nerve tissue. Only second to the consideration of the cell is the treatment of the tissues, and to say that it is most satisfactory is only half stating the facts.

In part fourth the organs are taken up, twelve chapters being devoted to their consideration. The circulatory system comes first, in which the bloodvessel and lymphvessel systems are clearly described in structure and development. The relations of small artery and vein are made plain by a diagram that is unsurpassed in lucidity.

The lymphatic organs become the subject of the second chapter and are described in general and in particular. The third chapter considers the skeletal system, the development and structure of bone being most interesting and is, withal, beautifully illustrated. The digestive system is considered at length in the next chapter. Then follow in this order the urinary system, the reproductive system, the skin and its appendages, the nervous system, and, finally, in chapter twelve, the organs of special sense are dealt with.

We have alluded before to the quality of the illustrations and must say, again, that their perfection is one of the charms of the book, and this fact, considered in relation to its general mechanical execution,—paper, printing, and complete make-up,—contributes toward the construction of one of the most substantial of the recent additions to medical literature.

The Practical Medicine Series of Year Books. Ten volumes. Issued under the general editorial charge of Gustavus P. Head, M.D., Professor of Laryngology and Rhinology in the Chicago Post-Graduate Medical School. Chicago: The Year Book Publishers. 1904. (Price, $1.00; entire series, $5.50, payable in advance.) Volume VIII. Materia Medica, Therapeutics, etc. Edited by George F. Butler and Collaborators. Volume IX. Physiology, Pathology, etc. Edited by W. A. Evans and Collaborators. Volume X. Skin and Venereal Diseases; Nervous and Mental Diseases. Edited by W. L. Baum and Hugh T. Patrick.

I. The contents of volume eight deal, first, with materia medica and therapeutics, by George F. Butler and George S. Browning; second, with preventive medicine, by Henry B. Favill; third, with climatology, by Norman Bridge; fourth, with suggestive therapeutics, by Daniel R. Brower; and, fifth, forensic medicine, by Harold N. Moyer. Nearly one-half the book is taken up with the first subject, though the editor confesses to a paucity of commendable work in therapeutics, unless *x*-ray and radium fields are excepted. The literature of all the topics has been well gleaned and prepared for professional use.

II. Physiology, pathology, bacteriology, anatomy, and a dictionary of new medical terms make up the contents of volume nine. Anatomy and pathology are supplied by W. A. Evans; physiology and bacteriology, by Adolph Gehrmann; and the dictionary of new words, by William Healy. Pathology is the largest section of the volume, unless it be bacteriology. Professor Evans has noted the essential features of the year for pathology, and Professor Gehrmann those pertaining to bacteriology.

III. The tenth and concluding volume of the year books for 1904 deals with skin and venereal diseases, by W. L. Baum; and nervous and mental diseases, by Hugh T. Patrick, the latter being assisted by Charles L. Mix. Patrick has made his topics interesting by a number of good illustrations, as, indeed, has Baum.

This entire series of year books for 1904 has been of excellent quantity and quality, giving the cream of medical literature in abstract, and furnishing a summary thereof at very reasonable cost.

---

Annual Report of the Surgeon-General of the Public Health and Marine Hospital Service of the United States. For the fiscal year 1904. Walter Wyman, M.D., Surgeon-General. Washington: Government Printing Office.

The annual reports of Surgeon-General Wyman are always full of interest. He presides over the most important disease preventing department that exists anywhere in the world, and these reports give in detail the results obtained.

The book before us deals with the medical inspection of immigrants in an important way, showing that 840,714 were inspected during the period covered by the report. Officers of the service are stationed at all ports where aliens seek admission to make examinations on call of the immigration service. Officers also have been sent to some foreign ports with a view to prevent prospective immigrants with prohibitive diseases from leaving for the United States. This latter plan works a great saving to the government as well as to the steamship companies and, besides, avoids the danger of importing infectious diseases to a considerable extent.

It would be well for the government to establish similar inspection at all European ports from which immigrants embark for our Atlantic ports, but as yet this has been possible only at Naples. The immigration problem presents many difficult propositions, not all of which are capable of solution off hand, but the restriction as to the importation of infectious diseases cannot be made too rigid.

The national quarantine service is of great importance and is directly related to the exclusion of epidemic disease. There are forty inspection stations, quarantine stations, at which service is rendered by the officers of this department. There are also land quarantine stations along the Mexican border. It is impossible to estimate the value of this method of excluding epidemic and infectious diseases from our domain, nor can the enormous amount of work required to carry it on efficiently be easily comprehended.

The report is full of interest as well as instruction to every person interested in preventing disease and in improving the sanitary conditions of our country.

---

Twenty-third Annual Report of the State Department of Health of New York. For the year ending December 31, 1902. With maps. Daniel Lewis, M.D., Commissioner. Albany: The Argus Company. Printers.

In the year 1902, according to this report, the total number of deaths from all causes was 124,160, the death rate being 17 in each thousand of population. While this rate may not appear to be a high one when compared with other geographical divisions of the Union, it is, nevertheless, larger than it should be, as explained by the commissioner in the statement that the mortality from preventable diseases was 14 per cent. of the total, which was above the average of preceding years. It is explained that the increase was due, at least in a considerable degree, to the prevalence of smallpox in many parts of the state, which disease was fatal to 442 persons during the year.

It is stated, again, that the deaths from typhoid fever were 1,318, less by 300 than the average for five years, but still too large a number from a water borne disease,—one indeed quite preventable. We regard it as among the first duties of the state to prevent the pollution of potable waters.

The laboratories of the health department are mentioned in considerable detail in the report and afford interesting topics for consideration. The cancer laboratory, located at Buffalo, deserves special consideration. A fine building, called the Gratwick laboratory, named for its donor, has been constructed and was put in operation some years ago. Several preliminary reports relating to the progress of its work have been issued, and there is encouragement that definite results will follow at no distant day.

This report contains much information concerning the sanitary conditions of many of the towns and villages throughout the state and is accompanied by two volumes of maps.

---

Progressive Medicine. Volume VI., December, 1904. A Quarterly Digest of Advances, Discoveries and Improvements in the Medical and Surgical Sciences. Edited by Hobart Amory Hare, M.D., Professor of Therapeutics and Materia Medica in the Jefferson Medical College of Philadelphia. Octavo, 374 pages. Illustrated. Philadelphia and New York: Lea Brothers & Company. (Per annum, in four cloth-bound volumes, $9.00; in paper binding, $6.00, carriage paid to any address.)

This number of a most valuable compend contains articles on diseases of the digestive tract and allied organs,—liver, pancreas, and peritoneum; anesthetics, fractures, dislocations, amputations, surgery of the extremities, and orthopedics; genitourinary diseases, diseases of the kidneys; and a practical referendum on therapeutics. The contributors are William T. Belfield, Joseph C. Bloodgood, John Rose Bradford, H. R. M. Landis, and J. Dutton Steele.

The book is full of practical suggestions, both medical and surgical, and is a useful reinforcement to the physician's literary armamentarium. Moreover, the price is reasonable, while the mechanical work is substantial. This volume, too, is well illustrated, a feature that adds not a little to the usefulness as well as attractiveness of this book.

---

A Textbook of Practical Therapeutics, with Especial Reference to the Application of Remedial Measures to Disease and their Employment upon a Rational Basis. By Hobart Amory Hare, M.D., B.Sc., Professor of Therapeutics and Materia Medica in the Jefferson Medical College, Philadelphia. With special chapters by G. E. de Schweinitz, Edward Martin, and Barton C. Hirst. Tenth edition, enlarged, thoroughly revised. Octavo, 908 pages, with 113 engravings and 4 full-page colored plates. Philadelphia and New York: Lea Brothers & Company. 1904. (Price: cloth, $4.00; leather, $5 00; half morocco, $5.50 net prices.)

Inasmuch as this book has been reviewed so many times in these columns and, moreover, as our opinion concerning its qualities has been expressed over and over again, it hardly becomes necessary to do more now than direct attention to the general fact that, in a little more than ten years, ten editions of the book have been printed; that each edition has received careful revision by the author; that every discovery of value in therapeutics, every new remedy of merit, and every useful method of treatment has been incorporated in this edition; and that illustrations have been employed to sharpen the already incisive text whenever such necessity has appeared.

There is no work on this topic with which we are familiar, that so nearly approaches perfection or so adequately meets the requirements of the everyday doctor who must needs combat disease and do it now, as does this treatise. Hare has placed the student and practitioner in his debt beyond liquidation in the work he has done in writing this book and in revising so carefully its ten successive editions, thus keeping it in the front line of progress.

---

A Manual of Personal Hygiene. By American Authors. Edited by Walter L. Pyle, A.M., M.D., Assistant Surgeon to the Wills Eye Hospital, Philadelphia. Second edition, revised and enlarged. Duodecimo, 441 pages. Illustrated. Philadelphia, New York, London: W. B. Saunders & Company. 1904. (Bound in silk, $1.50 net.)

When the first edition of this book was put forth it attracted the attention of many persons interested in personal hygiene other than physicians. It is gratifying that a second edition is demanded so soon, for it indicates a growing interest in a subject that is near the hearthstone of every household, one that invades every family circle, one that relates to the good health and happiness of every individual. It is an encyclopedic work, eight contributors besides the editor being concerned in its authorship. Charles G. Stockton, of Buffalo, who presents the hygiene of the digestive apparatus, sets the pace in the first section of the book, while the others make a gallant struggle for second place.

The additions in this issue include an illustrated system of home gymnastics, a chapter on domestic hygiene, and an appendix containing instruction in the simpler methods of hydrotherapy, thermotherapy, and mechanotherapy, together with a section on first aid in surgical accidents and emergencies.

Every teacher of the young should be familiar with the contents of this book which, for the most part, is free from technicalities, and is adapted to the schoolroom as well as domestic circle. If there is a physician inconversant with its teachings, he should make all haste to get the book and read it with care.

---

Appendicitis and Other Diseases about the Appendix. By Bayard Holmes, B.S., M.D., Professor of Surgery in the University of Illinois, Chicago. Three hundred and fifty pages. New York: D. Appleton & Company. 1904.

The discussion in regard to appendicitis is still going on and is likely to do so for some time to come. So many patients requiring surgical treatment are neglected, that it becomes the province of every surgeon to contribute what he may toward correcting this evil. Each monograph by an experienced man contributes to this end. The one before us cannot fail to aid in making a solution of many of the difficulties presented by appendicitis.

Holmes in his preface very appropriately says: "The practising physician is apt to be hopeful in his prognosis, no matter how grave the symptoms may be, or however inexplicable the symptom complex may appear."

In a group of adages at the close of the volume he also says: "The indications for treatment of appendicitis are simple. The appendix should be removed and the removal should be done at once."

It would be well for general practising physicians to obtain this monograph and study it carefully as it contains many maxims it would be well for them to heed.

Besides appendicitis, a number of other subjects are considered, such as peritonitis, intussusception, perforating typhoid ulcer and carcinoma of the intestinal tract. Appendicitis, however, is the principal topic and takes up more than four-fifths of the book. The volume contains a number of excellent illustrations some of which are in color. It were well if it were bound in cloth as frail paper is so destructive as to render the book almost useless after a little time.

---

International Clinics. A Quarterly of Illustrated Clinical Lectures and especially prepared articles on Treatment, Medicine, Surgery, Neurology, Pediatrics, Obstetics, Gynecology, Orthopedics, Pathology, Dermatology, Ophthalmology, Otology, Rhinology, Laryngology, Hygiene and other topics of interest to students and practitioners. By leading members of the medical profession throughout the world. Edited by A. O. J. Kelly, A.M., M.D., Philadelphia. Volume IV. Fourteenth series. 1905. Philadelphia: J. B. Lippincott Company. (Cloth, $2.00.)

The contents of this volume embrace contributions on treatment, four articles; on medicine, six articles; on surgery, seven articles; on gynecology, one article; on neurology, one article; and on pathology, two articles. The illustrations are numerous and instructive. This is true in a special sense of Dr. E. H. Bradford's article on lateral curvature of the spine, which is illuminated with forty half-tone engravings that serve to stamp it with great importance; indeed, it may be said to be the most instructive contribution lately made to the subject.

The entire volume bears the impress of careful preparation and is a fit companion to its predecessors.

---

A Laboratory Manual of Human Anatomy. By Lewellys F. Barker, M.B., Professor of Anatomy in the University of Chicago and Rush Medical College. Octavo, pp. 583. Illustrated. Philadelphia and London: J. B. Lippincott Company. 1904. (Price, $5.00.)

It is difficult to understand why this book should not be found in the hands of every student and teacher of anatomy. It contributes to the more systematic study and to the more perfect instruction, in a topic that must be mastered by every novitiate in medicine; mastered, too, at the very threshold of his collegiate career.

This manual,—and it does not assume to be anything more,—employs the nomenclature formulated by the German society of anatomists, hence is quite abreast of that adopted by the best English as well as American laboratory teachers. Old terms, however, which differ from the new, have been added in parenthesis, to prevent embarrassment on the part of those who are only familiar with the older textbooks and atlases.

It contains nearly 300 illustrations that depict in drawings and plates the more important structures of the human body. The most approved sources, including Spalteholz and Toldt, have been drawn upon with a liberal hand. Barker has rendered valuable service to the present tutors and pupils of anatomy that will reflect to his credit in all future time.

---

The American Yearbook of Medicine and Surgery for 1905. A Yearly Digest of Scientific Progress and Authoritative Opinion in all branches of Medicine and Surgery, drawn from journals, monographs, and textbooks of the leading American and foreign authors and investigators. Under the editorial charge of George M. Gould, A.M., M.D. In two volumes. Volume I, general medicine; volume II, general surgery. Two octavos of about 700 pages each, fully illustrated. Philadelphia and London: W. B. Saunders & Company. 1905. (Per volume: cloth, $3.00 net; half morocco, $3.75 net.)

The American yearbook has become so well established and thoroughly known to physicians, that the mere announcement of its issue from the press is sufficient to create an exhaustive demand for it. The present edition is constructed on the same lines that have characterised the issues of previous years. It is in two volumes, one being devoted to the consideration of general medicine, and the other embraces the field of general surgery.

In these two books is to be found everything of importance that has appeared in the literature of medicine and surgery during the year which it spans; moreover, it is so arranged as to be easily consulted. It is a valuable digest of the progress made for a year, with which through its aid a busy physician may keep step.

---

Blakiston's Quiz Compends. A Compend of Medical Latin. By W. T. Saint Clair, A.M., Professor of the Latin Language and Literature in the Male High School of Louisville, Ky. Duodecimo, 131 pages. Second edition, revised. Philadelphia: P. Blakiston's Son & Company. 1904. (Price, $1.00.)

It is important for every medical student to possess a knowledge of Latin; especially should he become familiar with medical Latin. In case he is not a classical scholar this compend will prove useful to him. By studying it carefully under a competent tutor it will serve him all through life, and render subsequent reading of medical books and journals much easier. Moreover, it is an excellent reference book for the student of medical literature to possess; it will enable him to refresh his memory upon forgotten points of doubtful word construction. A compend of this kind is capable of doing much good and its publication should be encouraged.

---

Transactions of the American Otological Society. Thirty-seventh Annual Meeting held at Atlantic City, N. J., July 11 and 12, 1904. F. L. Jack, M.D., Secretary. Published by the Society.

Several subjects of great interest were discussed at the meeting, the proceedings of which are reported in this volume. Otitis media came in for its full share of mention and elaboration, while osteomyelitis of the temporal bone was presented in two papers,—one by Charles W. Richardson, and one by Edward Bradford Dench.

The details of two important cases of chronic otitis media are given by Wendell C. Philips, in which the radical operation was made. In one the patient had tubercular hip joint disease and inherited syphilis; tubercular meningitis followed the operation, and finally death ensued. In the other, the operation was immediately followed by leptomeningitis and death. An interesting discussion was held on Dr. Philips's paper in which all the leading members participated.

The volume, full of instructive material, marks the progress of otological science and will be read by every otologist with attentive interest.

